# Osteoporosis in Patients With Respiratory Diseases

**DOI:** 10.3389/fphys.2022.939253

**Published:** 2022-07-12

**Authors:** Yue Ma, Shui Qiu, Renyi Zhou

**Affiliations:** ^1^ Department of Pulmonary and Critical Care Medicine, Shengjing Hospital of China Medical University, Shenyang, China; ^2^ Department of Orthopedics, First Hospital of China Medical University, Shenyang, China

**Keywords:** osteoporosis, respiratory disease, COPD, asthma, lung cancer, tuberculosis

## Abstract

Climate change, environmental pollution, and virus epidemics have sharply increased the number of patients suffering from respiratory diseases in recent years. Prolonged periods of illness and drug use increase the occurrence of complications in these patients. Osteoporosis is the common bone metabolism disease with respiratory disturbance, which affects prognosis and increases mortality of patients. The problem of osteoporosis in patients with respiratory diseases needs more attention. In this review, we concluded the characteristics of osteoporosis in some respiratory diseases including COPD, asthma, COVID-19, tuberculosis, and lung cancer. We revealed that hypoxia was the common pathogenesis of osteoporosis secondary to respiratory diseases, with malnutrition and corticosteroid abuse driving the progression of osteoporosis. Hypoxia-induced ROS accumulation and activated HIF-1α lead to attenuated osteogenesis and enhanced osteoclastogenesis in patients with respiratory diseases. Tuberculosis and cancer also invaded bone tissue and reduced bone strength by direct infiltration. For the treatment of osteoporosis in respiratory patients, oral-optimized bisphosphonates were the best treatment modality. Vitamin D was a necessary supplement, both for calcium absorption in osteogenesis and for improvement of respiratory lesions. Reasonable adjustment of the dose and course of corticosteroids according to the etiology and condition of patients is beneficial to prevent the occurrence and development of osteoporosis. Additionally, HIF-1α was a potential target for the treatment of osteoporosis in respiratory patients, which could be activated under hypoxia condition and involved in the process of bone remodeling.

## Introduction

Osteoporosis is a common disease characterized by decreasing bone mass, increasing bone fragility, and weakening bone microarchitecture (Kimmel et al., 2022). Under physiological conditions, bone tissue is in a dynamic balance between the bone formation effect of osteoblasts and the bone resorption effect of osteoclasts (Teissier et al., 2022). The direct pathogenesis of osteoporosis is that the weakened bone formation is difficult to compensate for the reduced bone mass of bone resorption (Yang et al., 2022). With aging, the prevalence of osteoporosis is increasing year by year (Dong et al., 2022). Complications of osteoporosis are an important factor threatening the health of patients. The incidence of osteoporotic fractures is close to ten percent, and the reduction in life expectancy of patients with osteoporotic fractures exceeds that of cancer in some regions (Johnell and Kanis, 2006; Wang et al., 2021a). The weak awareness of people’s regular physical examination and the insidious characteristics of osteoporosis have made this phenomenon worse. Early diagnosis and prevention of osteoporosis in high-risk groups will contribute to improving this public health problem.

Osteoporosis in high-risk groups mainly includes postmenopausal women with primary osteoporosis and diabetic patients with secondary osteoporosis (Cakmak et al., 2015; Ramchand et al., 2021). Endocrinology and orthopedic doctors paid more attention to these two groups of people to improve treatment efficiency. However, another high-risk group is attracting the attention of clinicians and researchers. Bone mineral density in patients with respiratory disease was significantly lower than that in the normal population (Chiu et al., 2021; Yadav et al., 2021). Patients with severe chronic obstructive pulmonary disease (COPD) were at four times the risk to develop osteoporosis (Bitar et al., 2021). The fracture ratio increased significantly in influenza patients after discharge (Axelsson et al., 2022). Drug application in the asthma treatment process also had a potential influence on the bone mass of patients (Chalitsios et al., 2022). These clinical data indicated that the patients with respiratory diseases had a high risk of getting osteopenia and osteoporotic fracture. It is essential to monitor bone mass changes in this high-risk group to prevent the occurrence of osteoporosis complications.

Hypoxia and drug abuse were the main causes of respiratory disease-induced osteoporosis (Zhuang et al., 2020; Sood et al., 2021). In recent years, the immune response induced in infected patients by the COVID-19 epidemic is also considered to be an important factor in enhancing RANKL-mediated bone resorption (Yang et al., 2021). Air pollution, smoking, viral infections, and cancer play a role in increasing the number of people suffering from respiratory diseases. Bone quality changes and bone mass detection in this population need more attention. Osteoporosis secondary to respiratory disease is becoming a research hotspot. In this review, we aimed to conclude the pathogenesis and characteristics of osteoporosis secondary to various respiratory diseases, reveal the influence of hypoxia on bone metabolism, and provide the opinion on the bone mass maintenance in treatment.

### Dyspnea and Hypoxia

Dyspnea was the common essential feature of respiratory diseases. A hypoxic state due to pulmonary ventilation and ventilation dysfunction was the fundamental cause of bone metabolism disorder in patients with respiratory diseases (Evans and Morgan, 2014).

Decreasing osteogenic marker expression could be observed under osteogenic induction in the hypoxia state (Teh et al., 2021). Hypoxia culture also promoted osteoclast differentiation by activating the NF-κB ligand and macrophage colony-stimulating factor (Kondo et al., 2021). ROS was a necessary means of hypoxia to cause pathological changes. Stress response enhancement and antioxidant function reduction occurred in cells under hypoxia conditions, leading to the production of ROS (Estrada-Cárdenas et al., 2022). The imbalance of bone metabolism caused by hypoxia was also due to the accumulation of ROS (Shao et al., 2022). ROS suppressed osteogenesis and mineralization of bone marrow mesenchymal stem cells (Huang et al., 2012). Meanwhile, ROS could initiate the mitochondrial apoptosis pathway of osteoblasts by increasing the expression of cleaved caspase-3 and the secretion of cytochrome c (Cao et al., 2015). Both activity and differentiation of osteoblasts were suppressed by ROS produced by hypoxia. As for osteoclasts, the programmed death of osteoclasts was significantly reduced under hypoxia conditions (Ni et al., 2021). Intramitochondrial ROS was associated with the differentiation of osteoclasts induced by hypoxia (Srinivasan et al., 2010). ROS participated in the autocrine process of osteoclasts by increasing the expression of cathepsin K and tartrate-resistant alkaline phosphatase to promote osteoclastogenesis (Srinivasan and Avadhani, 2007). Additionally, hypoxia led to inflammatory bone imbalance (Doi et al., 2022). Mitochondria were ineffective, and the glucose metabolism was dominated by anaerobic respiration under hypoxic conditions. The mechanism of hypoxia-induced inflammation was mainly ROS accumulation and lactate production, which were negative for bone formation (Hou et al., 2017).

HIF-1α was a necessary factor-mediated hypoxia-induced osteoporosis (Miyamoto, 2016). The effect of HIF-1α on bone metabolism was bidirectional (Song et al., 2020). On the one hand, HIF-1α assisted bone tissue had to adapt to the hypoxia microenvironment (Camacho-Cardenosa et al., 2020; Wang et al., 2022). HIF-1α overexpression was positive to prevent osteoblast apoptosis under pathological conditions (Xu et al., 2021). Activation of the HIF-1α signaling pathway contributed to bone formation (Jiang et al., 2020). On the other hand, excessive hypoxic stimulation was an activator of bone resorption. HIF-1α could increase the expression of RANKL (Zhu et al., 2019). Also, HIF-1α was associated with the activation of nuclear factor-activated T cells c1 (NFAc1) that was positive in osteoclast differentiation (Xiao et al., 2020). For treatment, intermittent hypoxic therapy could activate the adaptive process of hypoxia mediated by HIF-1α, which was helpful for the treatment of hypoxia-induced osteoporosis (Bergholt et al., 2021; Chen et al., 2021). Precisely, regulating the physiological function of HIF-1α in osteoblasts and osteoclasts might be a potential means to maintain bone homeostasis.

### COPD

COPD is the respiratory disease most closely associated with the development of osteoporosis. A previous study indicated that osteopenia or osteoporosis occurred in 50–70% COPD patients (Pobeha et al., 2010). Bone mineral density decreased significantly causing the prevalence of osteoporosis or vertebral compression fracture achieving 24.6% in COPD patients (Kakoullis et al., 2022), and 43.7% COPD patients had insufficiency of vertebral deformity and soft tissue lesions (Kupeli et al., 2021). Also, restricted thoracic diastolic function in osteoporosis patients suppressed respiratory function and aggravated COPD (Chen et al., 2018a). In animal experiments, the pulmonary emphysema-mediated COPD mouse model showed an obvious decrease of bone mass, which demonstrated the direct link between COPD and osteoporosis (Tsukamoto et al., 2019).

The mechanism of osteoporosis secondary to COPD was complicated. Chronic hypoxia, malnutrition, and glucocorticoid application were thought to be triggers for decreased bone mineral density (Fouda et al., 2017; Johansen et al., 2018; Caramori et al., 2019). Based on bone metabolism monitoring in COPD patients, researchers revealed that these patients had a lower capacity for bone formation with the increasing COPD severity (Tsukamoto et al., 2020). After COPD induction, the bone mineral content and femoral strength were less (Shea et al., 19852003). Percent forced expiratory volume in 1 s (%FEV_1_) was moderately correlated with the osteogenic markers including alkaline phosphatase and procollagen type 1 N-terminal propeptide (P1NP). With the deterioration of lung function and exacerbations of COPD, vitamin D decreased significantly (Calle Rubio et al., 2022). Vitamin D deficiency was also a potential factor of COPD-induced osteoporosis by inhibiting the absorption of calcium to suppress bone matrix formation. Kokturk et al. (2018) concluded an effective prediction method of osteoporosis diagnosis in COPD based on these osteogenic markers and revealed that Vitamin D and osteocalcin were more sensitive indicators (Masik et al., 2021). In terms of bone resorption, a study showed that activated neutrophils increased the contents of RANKL in COPD patients’ blood and COPD patients had a higher concentration of CrossLaps, both of which represented increased bone loss (Kochetkova et al., 2002; Hu et al., 2017). The pathological state of oxidative stress and inflammation in COPD patients was the enhancer of osteoclastogenesis (Stanojkovic et al., 2013). Inflammatory mediators IL-2 and IFNγ were inversely correlated with bone metabolism in COPD (Bon et al., 2010). Skeletal muscle dysfunction mediated by inflammation was also an important factor in COPD-induced osteoporosis (Zhang and Sun, 2021). High expression of IL-6 in COPD patients disrupted muscle–bone crosstalk and inhibited the secretion of irisin from muscle, which was positive for bone formation.

Among the patients with COPD, smokers are a more noteworthy group. Lung-specific changes in smokers were associated with the morbidity and mortality of COPD-induced osteoporotic fracture (Bon et al., 2020). Smoking mediated tracheal deformity, and emphysema increased the decrease of bone mineral density (Pompe et al., 2016). Therefore, it is necessary to detect bone mass in COPD patients, especially smokers. Unfortunately, previous studies had reported the drug treatment data of many secondary osteoporosis cases but COPD (Lehouck et al., 2010; Okazaki, 2015). Osteoporosis secondary to COPD needs more attention. Effective prevention of bone loss in patients with COPD contributed to the treatment of COPD and osteoporosis.

### Asthma

Asthma is an inflammatory respiratory disease characterized by airway hyperresponsiveness. Compared with COPD, asthma patients should scientifically detect the development of osteoporosis because asthma-induced dyspnea combined with osteoporosis could easily cause shock in patients. The mechanism of asthma-associated bone mass loss could be divided into asthma-induced osteoporosis and corticosteroid-induced osteoporosis, which was dominated by the latter. The direct effect of asthma leading to osteoporosis is mainly due to the occurrence of inflammation. The main pathogenesis of asthma was neutrophil-mediated inflammation. TNF-α participated in the process of neutrophil migration, which was associated with a decrease in bone mineral density (Shmarina et al., 2013). TNF-α was an inhibitor of osteogenesis and an activator of osteoclastogenesis (Han et al., 2022; Pramusita et al., 2022). TNF-α disrupted the balance of RANKL/OPG, resulting in the enhancement of bone resorption rather than formation (Wan et al., 2016). Additionally, IL-6 was also regarded as an important inflammatory factor involved in neutrophil-inflammation-mediated osteoporosis in asthma patients (Leng et al., 2005). Overexpression of IL-6 inhibited the differentiation of osteoblasts and obviously increased the expression of RANKL to increase osteoclast activity (El Kholy et al., 2018; Di Pompo et al., 2021).

Clinicians paid more attention to the problem that asthma patients were susceptible to osteoporosis and the prescription rates of bone mineral density testing and bisphosphonates therapy for osteoporosis in asthma patients were increasing (Soen et al., 2021). Enough physical activity and calcium intake were important for these patients. Meanwhile, they should rationally choose the treatment drugs according to the changes of blood and urinary calcium levels (Picado and Luengo, 1996). Bisphosphonates were the first choice for most of the asthma patients with decreased bone mass (Chalitsios et al., 2020). Vitamin D supplementation was also an effective method to prevent asthma-induced osteoporosis (Awadh et al., 2003).

### Corticosteroid-Induced Osteoporosis During COPD and Asthma Treatment

Osteoporosis commonly occurred during the treatment of COPD and asthma with corticosteroids (Chen et al., 2018b; Sood et al., 2021). Oral corticosteroids increased the risk of osteoporosis by 1.56–3.38 times in patients, and the risk would increase significantly with the cumulative dose of corticosteroids (Osteoporosis and fracture risk with, 2021). Corticosteroids decreased the trabecular bone score of the lumbar spine and hip, which increased the risk of fracture (Sandru et al., 2020). Some studies revealed that in nearly 50% of patients undergoing oral corticosteroid treatment exhibited risk of fractures (Chalitsios et al., 2021a). Unexpectedly, inhaled corticosteroid therapy might not appear to increase the risk of osteoporosis (Chalitsios et al., 2021b; Grosso et al., 2021).

As for corticosteroid-induced bone loss, some prospective observational studies revealed that long-term oral corticosteroids decreased the mineralization of bone tissue (Park et al., 2015). Osteocalcin was usually lowered with corticosteroid intake (Puolijoki et al., 1992; Cesana‐Nigro et al., 2019), and biosynthesis of Collagen I was also interfered by corticosteroids (Puolijoki et al., 1996). These significantly decreasing osteogenic markers also reflected the weakness of osteoblasts with corticosteroid treatment (Brabnikova Maresova et al., 2013). At the cellular level, exogenous corticosteroid would combine with its receptor on bone tissue to impair the survival of osteoblasts and inhibit apoptosis of osteoclasts (Lee et al., 2021). For cross-talk between bone cells, a high concentration of corticosteroids increased the ratio of RANKL/OPG to enhance bone resorption. Additionally, corticosteroids mainly acted on the mitochondria of osteoblasts, leading to the accumulation of reactive oxygen radicals (ROS), and induced pathological necrosis (Wu et al., 2019a; Peng et al., 2021). Excessive ROS alternated the membrane potential of mitochondria and increased the expression of mitochondrial apoptotic proteins including Bax, caspase 9, caspase 3, and cytochrome C, which decreased the activity of osteoblasts. Corticosteroids also increased tartrate-resistant acid phosphatase from 5b to hyperactivate and prolonged the lifespan of osteoclasts (Sato et al., 2019; Chen et al., 2020). Activation of the MAPK, Akt, NF-κB, and cytoplasmic 1 signaling pathways contributed to osteoclast differentiation when corticosteroid promoted RANKL to combine with RANK (Lin et al., 2021).

Application of corticosteroid in COPD and asthma patients needed to be valued to avoid the development of osteoporosis. The guidance for corticosteroid-induced osteoporosis had been provided for groups of different ages (Weare-Regales et al., 2021). People younger than 40 years old need regular bone quality testing, and those older than 40 need bone-preserving treatment. Also, the researchers attempted to optimize the dose of corticosteroids and explored the new treatment method for asthma to avoid the side effects of traditional corticosteroid therapy (González-Barcala et al., 2021; Sood et al., 2021).

### COVID-19

In past studies, scientists had suggested that the development of osteoporosis is not directly related to pneumonia. However, many studies indicated that bone metabolism disorder, bone mineral density decreasing, and bone microarchitecture destruction occurred in patients infected with SARS-CoV2 during the last 3 years of the COVID-19 pandemic (Mi et al., 2021; Tsourdi et al., 2022). COVID-19 increased the fragility of bone tissue and the risk of fractures with increasing severity (Battisti et al., 2021). Thoracic bone mineral density appeared to correlate with the mortality in COVID-19 patients (Tahtabasi et al., 2021), and the mortality also increased in vertebral fracture patients with COVID-19 (di Filippo et al., 2021). Additionally, a study from Korea reported that COVID-19 vaccination also led to some endocrine diseases like osteoporosis (Ku et al., 2021). Detecting bone mineral density and preventing bone loss would contribute to increasing therapeutic effect of COVID-19 patients.

The causes of osteoporosis induced by COVID-19 was broad including inflammatory storm, home quarantine, lack of exercise, and glucocorticoid treatment (Napoli et al., 2020). SARS-CoV-2 competed for binding to and caused the deficiency of ACE2 in mesenchymal stem cells, which increased the expression of inflammatory factor TNF-α to enhance bone resorption mediated by RANK (Tao et al., 2020). Pro-inflammatory cytokines including IL-1β and IL-2 highly expressed in COVID-19 patients also involved in the process of osteoporosis. IL-1β and IL-2 were powerful cytokines to stimulate bone resorption by upregulating RANKL (Ruscitti et al., 2015; Momiuchi et al., 2021). Other highly expressed cytokines with SARS-CoV-2 infection such as IL-8 and IL-10 were associated with bone metabolism, accelerating the process of bone loss (Amarasekara et al., 2018; Carnovali et al., 2021). Also, SARS-CoV-2 infection could induce hypoparathyroidism, resulting in the disturbance of calcium and phosphorus metabolism (Elkattawy et al., 2020). Hypocalcemia and hyperphosphatemia were negative for bone homeostasis (Gittoes et al., 2020; Cheng and Kuo, 2022). Additionally, vitamin D was a crucial factor to predict the outcomes of COVID-19 and played an important role in the correlation of COVID-19 and osteoporosis (Amrein et al., 2021; Bassatne et al., 2021). COVID-associated coagulopathy interfered the interaction of Vitamin D and platelets, which drove the imbalance of bone remodeling (Salamanna et al., 2021), and the content of circulating 250-hydroxyvitamin D was lower in COVID-19 patients, which was negative for osteogenesis (Diaz-Curiel et al., 2021). Home quarantine and lack of exercise were also catalysts for the development of osteoporosis in the elderly (Kirwan et al., 2020). Reduced physical activity and variated diet habits decreased the muscle mass and weakened the protection of bone tissue. Low energy injuries increased the ratio of fractures in elderly population (Lv et al., 2020).

Significant changes appeared in the management of osteoporosis under the COVID-19 epidemic (Kong et al., 2021; Salvio et al., 2022). Clinical data demonstrated that anti-osteoporosis treatment did not increase the infective risk, severity, and mortality of COVID-19 (Atmaca et al., 2022). Oral bisphosphonates might be a good choice for osteoporosis patients with COVID-19 due to its anti-inflammation effect (Degli Esposti et al., 2021). Vitamin D supplementation was positive to prevent the occurrence and development of osteoporosis (Raisi-Estabragh et al., 2021; Ulivieri et al., 2021). In conclusion, oral medication and self-injectable anabolic agents were the mainstay of osteoporosis treatment during the COVID-19 pandemic (Sundaresh, 2020).

### Pulmonary Tuberculosis

Pulmonary tuberculosis was a potential risk for the development of osteoporosis (Choi et al., 2017). Tuberculosis patients had significantly higher odds of osteoporotic fractures (Yeh et al., 2016; Chen et al., 2017). Primary pulmonary tuberculosis mainly led to malnutrition and further induced decreased bone mass in patients (Rose et al., 1999; Ishii et al., 2007). Vitamin D deficiency played an important role in tuberculosis-induced osteoporosis (Shin et al., 2015). Vitamin D receptor was regarded as a linkage between tuberculosis and osteoporosis (Roy et al., 1999). Therapeutic method targeting Vitamin D might be effective to prevent the occurrence and deterioration of osteoporosis. Additionally, tuberculosis in bone tissue was mainly secondary to pulmonary tuberculosis. Tuberculosis disrupted the soft tissue around bone first when it invaded the skeletal system (Pattamapaspong et al., 2011). Muscle infection weakened the maintenance and prevention of bone tissue. Notably, bone tuberculosis was more likely to occur in parts with heavy load and high activity, which greatly increased the risk of fractures. In microenvironment, *Mycobacterium tuberculosis* could increase the ratio of RANKL/OPG to inhibit the activity of osteoblasts (Zhang et al., 2014; Zhang et al., 2015). IL-1 and TNF-α secretion induced by *Mycobacterium tuberculosis* also involved in the disorder of bone metabolism, leading to bone mass loss (Wright and Friedland, 2004; Yi et al., 2018). As for treatment, oral calcium and Vitamin D intake were positive for improving bone mass during tuberculosis (Shin et al., 2015).

### Lung Cancer

Lung cancer was the most dangerous factor of bone mineral density changes in respiratory diseases (Savula et al., 2004). Osteopenia and osteoporosis were more likely to occur in people with lung cancer (Huang et al., 2022). Among different types of lung cancer, non-small cell lung cancer was most associated with osteoporosis, and bone metastasis was also the risk factor for its negative prognosis (Rinaldi et al., 2019).

The results obtained from the serum metabolite measurement in lung cancer patients indicated decreasing of calcium and increasing of osteocalcin, leading to bone imbalance (Dumanskiy et al., 2018). Nuclear factor kappa-B mediated the effect of bone resorption enhanced by lung cancer cells (Ma et al., 2018). The BMP2 signaling and Wnt/β-catenin pathways mediated the bone invasion of lung cancer cells (Huang et al., 2021; She et al., 2021). Secondary bone tumor eroded bone substance, disrupted bone structure, and increased the risk of fractures (Niu et al., 2022; Sethakorn et al., 2022). Additionally, microRNAs might play an important role in the development of osteoporosis secondary to lung cancer (Bellavia et al., 2019). Some microRNAs provided the implantation sites for cancer cell metastasis on bone tissue, and exosomes miR-17-5p derived from non-small cell lung cancer was determined to be positive in osteoclast differentiation by targeting PTEN (Wang et al., 2021b). Osteolytic metastasis was also a great challenge for the patients with lung cancer (Wu et al., 2021a). Serum albumin decreasing threatened the homeostasis of bone tissue in these patients (Miyashita et al., 2021).

At present, low-dose chest computed tomography was verified to be effective for the diagnosis of osteoporosis in lung cancer patients (Wu et al., 2019b; Pan et al., 2020). Based on the test results, bone density was also an independent predictor of mortality (Buckens et al., 2015). For treatment, bisphosphonates were applied to treat bone cancer metastasized from the lung together with osteoporosis but with obvious side effects (Tao et al., 2018; Mbese and Aderibigbe, 2021). Osteoporosis medications were also regarded as potentially inappropriate medications for older patients with lung cancer (Ham et al., 2022). Interestingly, some anti-osteoporosis drugs had a therapeutic effect on lung cancer, which provide a reverse view for the treatment of osteoporosis in lung cancer patients (Almutairi et al., 2019; Wu et al., 2020). Also, vitamin D supplement was positive to maintain the calcium absorption for bone matrix formation (Laskowski et al., 2016). In a word, rational and effective treatment modalities with few adverse effects need to be developed further.

### Environmental Exposures to Pollutants

Environmental pollution was an important factor causing the decline of respiratory system function and leading to osteoporosis. Environmental pollutants related to the development of osteoporosis mainly included the particles with diameter <2.5 μm (PM_2.5_) and automobile exhaust (Chen et al., 2015; Pang et al., 2021). Previous studies reported that decreased spinal bone mineral density could be observed after exposure to PM_2.5_ (Ranzani et al., 2020). The calcaneus quantitative ultrasound index was lower with the increase of PM_2.5_ (Wu et al., 2021b). PM_2.5_ was positively correlated with serum RANKL and the osteoclast precursor CD14 (+) CD16 (+) monocytes (Saha et al., 2016). Additionally, PM_2.5_ could induce the secretion of inflammatory cytokines such as TNF-α, IL-1β, and IL-6, all of which were stimulators of bone resorption (Pang et al., 2021). Additionally, vitamin D was deficient with PM_2.5_ exposure (Afsar et al., 2019). Disturbance of calcium absorption without the assistance of vitamin D was not conducive to bone remodeling. The contents of automobile exhaust leading to the occurrence of osteoporosis were mainly SO_2_ and NO_2_ (Liu et al., 2021; Heo et al., 2022). Long-term exposure to SO_2_ and NO_2_ increased the risk of osteoporotic fractures (Mazzucchelli et al., 2018).

The prevention of osteoporosis induced by environmental pollutants was mainly to reduce the discharge of these pollutants. Controlling the use of energy containing sulfur and nitrogen compounds will contribute to reducing their oxidation, thereby preventing conversion to PM_2.5_. Bone mass monitoring is also necessary for high-risk groups living near factories or highways. Additionally, reasonable supplementation of supplements such as vitamin D and calcium tablets for these populations may be an effective measure to prevent bone loss.

## Discussion

As the climate changes and the environment deteriorates, the prevalence of respiratory diseases in the elderly is increasing (Aliyu and Botai, 2018; Peng et al., 2022). In recent years, the epidemic of COVID-19 is a major challenge to the barrier of the human respiratory system (Shivshankar et al., 2022). Oxygen supplement reduction and carbon dioxide accumulation caused by gas exchange efficiency decreasing disrupted the normal physiology in the patients with decreased lung function (Williamson et al., 1993). Additionally, the treatment cycle is relatively long and the probability of radical cure is extremely low for respiratory diseases compared to other diseases. Patients with respiratory disease often require prolonged clinical intervention and lifelong maintenance with medication. Therefore, pathological changes secondary to respiratory diseases and drug side effects are problems that cannot be ignored in the process of treatment.

**FIGURE 1:**
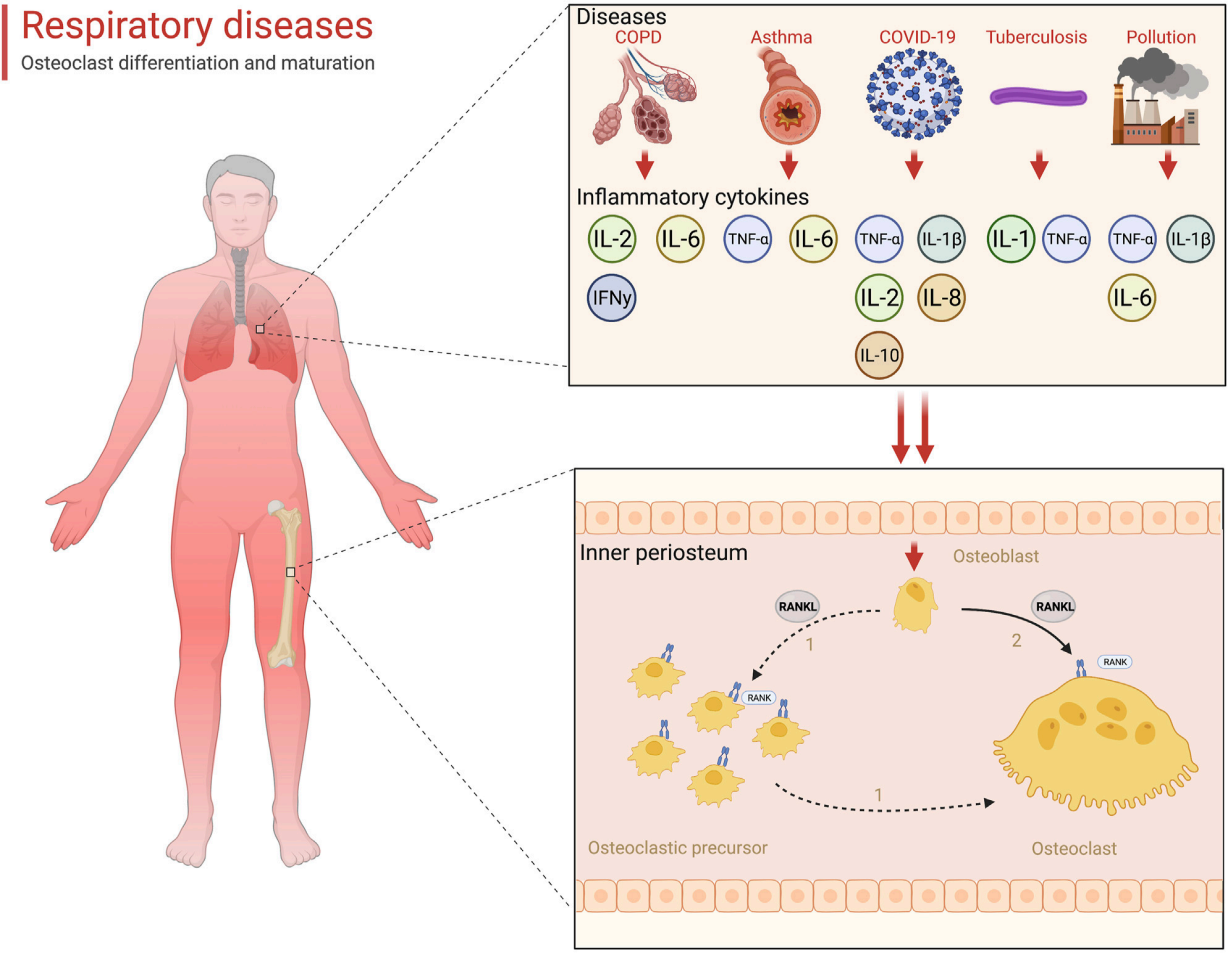
Inflammatory storm-mediated bone resorption with respiratory diseases (created with BioRender.com).

Skeletal health is valued in the clinical management of patients with respiratory diseases. Increased bone fragility and reduced flexibility decreased thoracic mobility, resulting in the limitation of breathing movements. The formation of this vicious circle greatly reduced the quality of life and increased the mortality rate of patients. Osteoporosis was the most common bone metabolism disease occurring in the respiratory patients. Respiratory diseases had been regarded as a high-risk factor of osteoporosis development (Leslie and Morin, 2020). In our review, we concluded several respiratory diseases that were predisposed to osteoporosis and revealed their pathogenesis. Hypoxia was the common problem. The essence of various respiratory diseases was the decline of lung function, which led to the gas exchange disorder in the lungs, leaving the body in a state of hypoxia. Hypoxia not only directly destroyed bone balance by increasing intracellular ROS but also induced inflammation, which further enhanced bone resorption (Halpin, 2007; Lin et al., 2020). Tuberculosis and tumors could erode bone tissue by metastasizing and causing bone mass loss (Coleman et al., 2014; Zeng et al., 2019). Inflammation cytokines were the accelerators for the development of osteoporosis in patients with respiratory diseases (Table 1). A variety of inflammatory factors are involved in the activation of RANKL (Figure 1). Additionally, prolonged breathing disturbances led to metabolic disorders that left patients in a state of malnutrition and affected the normal remodeling function of bone tissue (Ozcakir et al., 2020). Corticosteroid application in clinical treatment was also a non-negligible factor that threatens bone health.

**TABLE 1:** Inflammatory cytokines related to osteoporosis in different respiratory diseases.

Respiratory disease	Inflammatory cytokine
COPD	IL-2, IFNγ, and IL-6
Asthma	TNF-α and IL-6
COVID-19	TNF-α, IL-1β, IL-2, IL-8, and IL-10
Tuberculosis	IL-1 and TNF-α
Environmental pollution	TNF-α, IL-1β, and IL-6

With regard to the treatment of osteoporosis in patients with respiratory diseases, clinicians had accumulated a lot of data and experience. Bisphosphonates were currently the first choice of drugs for patients with respiratory diseases complicated with osteoporosis, but side effects were also obvious. Modifications to bisphosphonates were underway to improve treatment efficacy. Rational formulation of the dose and frequency of corticosteroid use according to the characteristics and severity of diseases could help prevent the occurrence of osteoporosis. Vitamin D was an essential supplement to improve bone mass for patients with respiratory diseases. Meanwhile, HIF-1α played an important role in hypoxia-induced osteoporosis and was directly involved in the regulation of bone homeostasis, which was a potential target for the treatment of osteoporosis in respiratory patients.
